# Association between aspirin and mortality in critically ill patients with atrial fibrillation: a retrospective cohort study based on mimic-IV database

**DOI:** 10.3389/fcvm.2024.1280149

**Published:** 2024-05-17

**Authors:** Meijuan Zhang, Yadong Zuo, Zhanquan Jiao

**Affiliations:** Department of Cardiology, Tianjin Haihe Hospital, Tianjin, China

**Keywords:** aspirin, mortality, critically ill patients, atrial fibrillation, Cox proportional hazard model

## Abstract

**Background:**

Atrial fibrillation (AF) is a prevalent issue among critically ill patients, and the availability of effective treatment strategies for AF is limited.

**Aim:**

The objective of this study was to evaluate the mortality rate associated with AF in critically ill patients who were either aspirin or non-aspirin users.

**Methods:**

This cohort study incorporated critically ill patients with AF from the Medical Information Mart for Intensive Care database. The study compared incidences of 28-day mortality, 90-day mortality, and 1-year mortality between patients with and without aspirin prescriptions. To assess the association between aspirin and the endpoints, Kaplan-Meier analysis and Cox proportional hazards regression analyses were conducted.

**Results:**

In this study, a total of 13,330 critically ill patients with atrial fibrillation (AF) were included, of which 4,421 and 8,909 patients were categorized as aspirin and non-aspirin users, respectively. The 28-day, 90-day, and 1-year mortality rates were found to be 17.5% (2,330/13,330), 23.9% (3,180/13,330), and 32.9% (4,379/13,330), respectively. The results of a fully-adjusted Cox proportional hazard model indicated that aspirin use was negatively associated with the risk of death after adjusting for confounding factors (28-day mortality, HR 0.64, 95% CI 0.55–0.74; 90-day mortality, HR 0.65, 95% CI 0.58–0.74; 1-year mortality, HR 0.67, 95%CI 0.6∼0.74). The results of the subgroup analysis indicate a more robust correlation, specifically among patients under the age of 65 and those without a history of congestive heart failure or myocardial infarction.

**Conclusions:**

The utilization of aspirin may exhibit a correlation with a reduction in risk-adjusted mortality from all causes in critically ill patients diagnosed with atrial fibrillation. However, additional randomized controlled trials are necessary to elucidate and confirm this potential association.

## Introduction

Atrial fibrillation (AF) is a prevalent arrhythmia in clinical settings (1)^,^ particularly in intensive care units (2)^,^ with an estimated global incidence of 46.3 million patients (3). AF is associated with an elevated risk of stroke and reduced cardiac function, which in turn increases the likelihood of mortality (4). Critically ill patients with either new or recurrent AF are at a higher risk of hospital mortality (5, 6). Despite the growing incidence and prevalence of AF, there is currently a lack of effective prevention and treatment strategies.

Aspirin is a fundamental component of antiplatelet therapy and is extensively employed in the management and prophylaxis of cardiovascular and cerebrovascular ailments (7, 8). In AF mice, aspirin administration impeded pathological atrial remodeling, mitigated fibrosis, and safeguarded mitochondrial function, thereby leading to a marked decrease in the occurrence of spontaneous AF. The authors inferred that aspirin holds considerable potential as a therapeutic approach for the primary prevention of AF (9).

The Real-World Data Study conducted on patients with non-valvular AF in the UK revealed that the aspirin group exhibited a lower all-cause mortality rate compared to the groups administered with other antiplatelet agents and those without AF prescription (10).

The mean annual healthcare costs of patients receiving aspirin were found to be the lowest. A meta-analysis examining the impact of antiplatelet therapy on mortality in sepsis patients revealed that aspirin was efficacious in reducing in-hospital or ICU mortality (11). However, a nested cohort study produced contrasting results, indicating that aspirin use in critically ill patients was not linked to lower mortality rates but rather an elevated risk of ICU-acquired severe sepsis, prolonged mechanical ventilation, and increased ICU length of stay (12).

However, the available evidence regarding the correlation between the utilization of aspirin and both immediate and prolonged mortality in critically ill patients with atrial fibrillation is restricted. As a result, the objective of our research was to examine whether there was an independent association between aspirin and mortality in critically ill patients with atrial fibrillation.

## Methods

### Study design and population

Critically ill patients with AF were recruited from the Medical Information Mart for Intensive Care (MIMIC)-IV (version 2.1), which is a publicly available real-world clinical database that includes ICU admissions at Beth Israel Deaconess Medical Center from 2008 to 2019 (13). Access to the database was granted to one of the authors (Meijuan Zhang, ID: 10784351).

This study involved the enrollment of intensive care unit patients who were diagnosed with atrial fibrillation using the International Classification of Diseases, Ninth and Tenth Edition diagnosis codes. The Declaration of Helsinki was adhered to during the course of this study. Given that the study was retrospective and all data were de-identified, informed consent and ethical approval were waived. The Strengthening the Reporting of Observational Studies in Epidemiology (STROBE) guidelines were utilized to report the findings of this study (14).

The inclusion criteria comprised of the following: (1) critically ill patients diagnosed with AF were deemed eligible; (2) solely adult patients aged over 18 years were included; and (3) the initial admission to the intensive care unit was taken into account in cases where a patient had multiple ICU admissions.

### Aspirin use

Aspirin use was defined as a record of aspirin prescription in MIMIC-IV database.

### Covariates

The study included following variables obtained at baseline: demographic characteristics, vital sign and creatinine, glucose, white blood count(WBC),hemoglobin, platelets, charlson comorbidity index, oxford acute severity of illness score (OASIS), acute physiological score III (APSIII), and simplified acute physiology score II (SAPSII), comorbidities (severe liver disease, malignant cancer, cerebrovascular disease, congestive heart failure, myocardial infarction), Medications[beta-blocker, amiodarone, digoxin, statin, warfarin, new oral anticoagulants(NOAC)], ventilation use, vasoactive.

### Outcomes

The present study delineates the comprehensive procedure for defining mortality risk, which entailed utilizing 28-day, 90-day, and 1-year mortality as the outcome events, extracted from the MIMIC-IV database. The monitoring of outcome events persisted for 365 days following admission to the ICU.

### Statistical analysis

The descriptive analysis was conducted on all participants. Mean and standard deviation or median and interquartile range were used to express continuous variables for normal and skewed distributions, respectively. To test differences among groups, categorical variables were analyzed using *χ*^2^, normal distribution using Student's independent *t*-test, and skewed distribution using Mann–Whitney *U* test. The mortality risk between groups was estimated using the Kaplan-Meier method (log-rank analyses).

The management of missing data was conducted based on the percentage of missing values. In the present study, proBNP and sequential organ failure assessment (SOFA) score were excluded due to the presence of missing values exceeding 30%.

We employed both univariate and multivariate Cox proportional hazard analysis, constructing four models: crude model, adjusted for no covariates. model 1, only adjusted for gender, age, marital status, insurance and race; model 2, model 1 plus heart rate, respiratory rate (RR), mean blood pressure (MBP), SPO2, creatinine, glucose, hemoglobin, platelets, WBC, ventilation use and vasoactive use; model 3, model 2 plus severe liver disease, malignant cancer, cerebrovascular disease, congestive heart failure, myocardial infarction, APSIII, SAPSII, OASIS, charlson comorbidity index, beta-blocker, amiodarone, digoxin, statin, warfarin and NOAC.

The study conducted sensitivity and subgroup analyses, wherein patients with prior aspirin use upon ICU admission were excluded as part of the sensitivity analysis. Furthermore, a subgroup analysis was performed using multivariable Cox proportional hazards regression analysis, with age, gender, severe liver disease, malignant cancer, cerebrovascular disease, congestive heart failure, myocardial infarction, warfarin and NOAC as the variables of interest. The study also evaluated the interactions between subgroups through the likelihood ratio test.

The statistical analyses were performed utilizing R 3.3.2 (http://www.R-project.org, The R Foundation) and Free Statistics software version 1.7. Two-tailed tests were executed, and statistical significance was determined by a *p*-value of less than 0.05 (two-sided).

## Results

### Baseline characteristics

**Figure 1:**
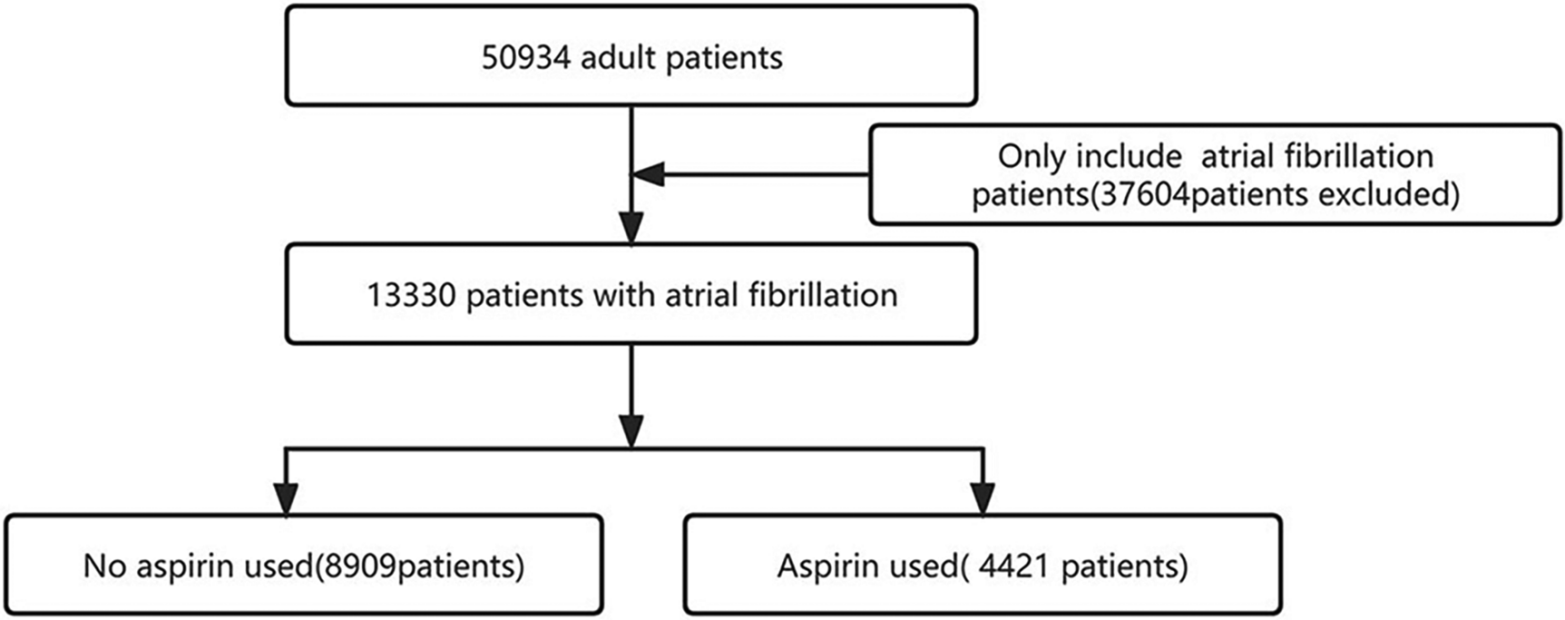
Selection of the study population.

We identified 13,330 individuals with their first admission to the ICU who developed AF (Figure 1). Of these patients, 4,421 (33%) used aspirin. Table 1 presents the baseline characteristics of the selected participant. In general, the average age of all selected participants was 73.7 ± 11.8 years, 7,793 (58.5%) were male, 9,886 (74.2%) were white people.

**Table 1:** Baseline characteristics of patients.

Variables	Total (*n* = 13,330)	No aspirin (*n* = 8,909)	Aspirin (*n* = 4,421)	*P*-value
Age (years), mean ± SD	73.7 ± 11.8	74.3 ± 12.1	72.5 ± 11.0	<0.001
Gender, *n* (%)				<0.001
Female	5,537 (41.5)	3,955 (44.4)	1,582 (35.8)	
Male	7,793 (58.5)	4,954 (55.6)	2,839 (64.2)	
Race, *n* (%)				<0.001
White people	9,886 (74.2)	6,692 (75.1)	3,194 (72.2)	
Other	3,444 (25.8)	2,217 (24.9)	1,227 (27.8)	
Marital status, *n* (%)				<0.001
Married	6,587 (53.6)	4,287 (51.9)	2,300 (57.1)	
Other	5,694 (46.4)	3,967 (48.1)	1,727 (42.9)	
Insurance, *n* (%)				<0.001
Medicare	7,965 (59.8)	5,444 (61.1)	2,521 (57)	
Medicaid	376 (2.8)	278 (3.1)	98 (2.2)	
Other	4,989 (37.4)	3,187 (35.8)	1,802 (40.8)	
Heart rate (bpm), mean ± SD	84.6 ± 16.2	85.4 ± 16.6	82.9 ± 15.0	<0.001
MBP (mmHg), mean ± SD	76.9 ± 10.5	76.8 ± 10.8	77.0 ± 10.0	0.356
RR (bpm), mean ± SD	19.4 ± 3.7	19.4 ± 3.7	19.2 ± 3.5	0.003
SPO_2_(%), mean ± SD	96.7 ± 2.4	96.6 ± 2.6	96.9 ± 1.9	<0.001
Scr(mg/dl), mean ± SD	1.1 (0.8, 1.7)	1.1 (0.8, 1.7)	1.1 (0.9, 1.6)	0.965
Glucose(mg/dl), mean ± SD	139.5 ± 43.9	139.3 ± 44.7	140.1 ± 42.3	0.329
WBC (×10^9^), median (IQR)	12.7 (9.2, 17.3)	12.2 (8.8, 16.6)	13.9 (9.9, 18.4)	<0.001
Hemoglobin (g/L), mean ± SD	10.1 ± 2.2	10.2 ± 2.2	9.8 ± 2.1	<0.001
Platelets(×10^12^), mean ± SD	179.8 ± 92.6	187.7 ± 97.6	163.9 ± 79.2	<0.001
OASIS, mean ± SD	33.5 ± 9.0	34.0 ± 9.2	32.4 ± 8.5	<0.001
SAPSII, mean ± SD	39.9 ± 13.0	40.5 ± 13.5	38.9 ± 11.8	<0.001
APSIII, mean ± SD	49.8 ± 23.4	51.5 ± 24.3	46.3 ± 21.1	<0.001
Charlson comorbidity index, mean ± SD	6.6 ± 2.5	6.6 ± 2.5	6.7 ± 2.5	0.003
Severe liver disease, *n* (%)	307 (2.3)	243 (2.7)	64 (1.4)	<0.001
Malignant cancer, *n* (%)	1,588 (11.9)	1,238 (13.9)	350 (7.9)	<0.001
Cerebrovascular disease, *n* (%)	2,417 (18.1)	1,614 (18.1)	803 (18.2)	0.947
Congestive heart failure, *n* (%)	6,008 (45.1)	3,807 (42.7)	2,201 (49.8)	<0.001
Myocardial infarction, *n* (%)	2,927 (22.0)	1,550 (17.4)	1,377 (31.1)	<0.001
Beta-blocker, *n* (%)	5,005 (37.5)	1,611 (18.1)	3,394 (76.8)	<0.001
Amiodarone, *n* (%)	2,086 (15.6)	421 (4.7)	1,665 (37.7)	<0.001
Digoxin, *n* (%)	681 (5.1)	275 (3.1)	406 (9.2)	<0.001
Statin, *n* (%)	4,001 (30.0)	914 (10.3)	3,087 (69.8)	<0.001
Warfarin, *n* (%)	2,200 (16.5)	535 (6)	1,665 (37.7)	<0.001
NOAC, *n* (%)	1,393 (10.5)	560 (6.3)	833 (18.8)	<0.001
Ventilation use, *n* (%)	5,177 (38.8)	3,185 (35.8)	1,992 (45.1)	<0.001
Vasoactive use, *n* (%)	5,985 (44.9)	3,662 (41.1)	2,323 (52.5)	<0.001
28-day mortality, *n* (%)	2,330 (17.5)	1,912 (21.5)	418 (9.5)	<0.001
90-day mortality, *n* (%)	3,180 (23.9)	2,533 (28.4)	647 (14.6)	<0.001
1-year mortality, *n* (%)	4,379 (32.9)	3,400 (38.2)	979 (22.1)	<0.001

Bpm, beat per minute; RR, respiratory rate; MBP, mean blood pressure; WBC, white blood count; APSIII, acute physiological score III; SAPSII, simplified acute physiology score II; OASIS, oxford acute severity of illness score; NOAC, new oral anticoagulants.

Aspirin users had the higher values in SPO2, WBC, Charlson comorbidity index, ICU stay, hospital stay, and consisted of more congestive heart failure, myocardial infarction, medications (beta-blocker, amiodarone, digoxin, statin, warfarin, NOAC, vasoactive) and ventilation use. The opposite patterns were observed in heart rate, RR, hemoglobin, platelets, OASIS, SAPSII, APSIII.

### Primary outcome

The 28-day mortality rates were 9.5% (418/4,421) and 21.5% (1,912/8,909) for aspirin and non-aspirin users, respectively. The 90-day (14.6%, 647/4,421) and 1-year mortality (22.1%, 979/4,421) of aspirin users were lower than those of non-aspirin users (28.4%, 2,533/8,909; 38.2%, 3,400/8,909).

This study involved the construction of four models to examine the independent impacts of aspirin on mortality, utilizing both univariate and multivariate Cox proportional hazard models. The resulting hazard ratios (HR) and corresponding 95% confidence intervals (CI) were presented in Table 2.

**Table 2:** Associations between aspirin and mortality risk of all participants.

Variables	28-day mortality			90-day mortality			1-year mortality		
	HR	95% CI	*P*	HR	95% CI	*P*	HR	95% CI	*P*
Crude model									
No aspirin	1	Ref		1	Ref		1	Ref	
Aspirin use	0.41	0.37–0.45	<0.001	0.47	0.43–0.51	<0.001	0.51	0.47–0.55	<0.001
Model 1									
No aspirin	1	Ref		1	Ref		1	Ref	
Aspirin use	0.43	0.39–0.49	<0.001	0.5	0.45–0.54	<0.001	0.55	0.51–0.59	<0.001
Model 2									
No aspirin	1	Ref		1	Ref		1	Ref	
Aspirin use	0.46	0.41–0.52	<0.001	0.52	0.47–0.57	<0.001	0.57	0.52–0.61	<0.001
Model 3									
No aspirin	1	Ref		1	Ref		1	Ref	
Aspirin use	0.64	0.55–0.74	<0.001	0.65	0.58–0.74	<0.001	0.67	0.6–0.74	<0.001

Crude Model: No adjustment.

Model I: Adjusted for age, gender, marital status, insurance and race.

Model II: Adjusted for variables in model Ⅰ plus heart rate, mean blood pressure, respiratory rate, SPO2, creatinine, glucose, hemoglobin, platelets, and white blood count.

Model III: Adjusted for variables in model Ⅱ plus severe liver disease, malignant cancer, cerebrovascular disease, congestive heart failure, myocardial infarction, APSIII, SAPSII, OASIS, charlson comorbidity index, beta-blocker, amiodarone, digoxin, statin, warfarin, NOAC, ventilation use and vasoactive use.

APSIII, acute physiological score III; SAPSII, simplified acute physiology score II; OASIS, oxford acute severity of illness score, NOAC, new oral anticoagulants.

The results of the multivariate Cox proportional hazard analysis indicate a significant reduction in 28-day mortality among individuals who use aspirin. The hazard ratio (HR) was 0.64 (95% CI 0.55–0.74, *P* < 0.001). Furthermore, the multivariate Cox proportional hazards regression analysis also demonstrated that aspirin use was associated with a decreased risk of 90-day and 1-year mortality (HR 0.65, 95% CI 0.58–0.74, *P* < 0.001; HR 0.67, 95% CI 0.6–0.74, *P* < 0.001).

**Figure 2:**
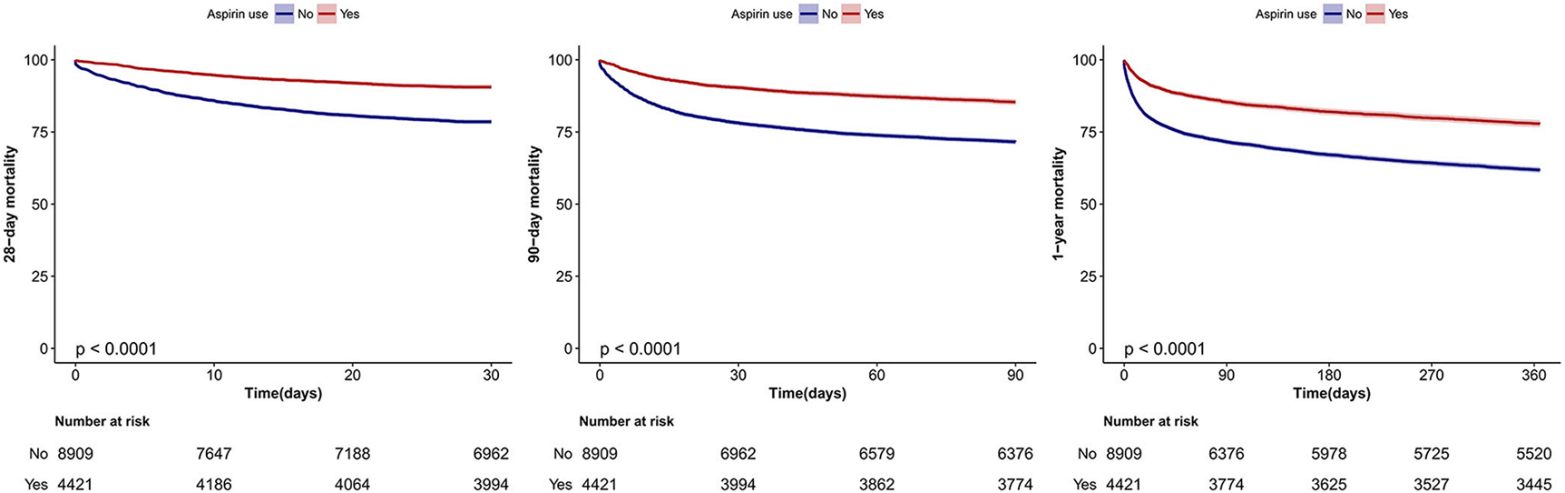
Kaplan–Meier survival curves for 28-day, 90-day and 1-year mortality of critically ill patients with atrial fibrillation. Log-rank test.

The results of the Kaplan-Meier curve analysis indicated that individuals who utilized aspirin had a decreased rate of mortality at 28-day, 90-day, and 1-year intervals (*p* < 0.01, as determined by the Log-rank test; see Figure 2).

### Sensitivity and subgroup analysis

Following the exclusion of 1,809 patients prescribed aspirin before admission to the ICU from the full cohort (*N* = 13,330), 11,521 patients remained. The observed associations between aspirin use and mortality risk remained consistent, as evidenced by hazard ratios of 0.54 (95% CI 0.44–0.65) for 28-day mortality, 0.56 (95% CI 0.49–0.66) for 90-day mortality, and 0.6 (95% CI 0.53–0.68) for 1-year mortality.

Individuals with severe liver disease or malignant cancer are likely to have contraindications to aspirin and a high mortality rate. We excluded these patients (*n* = 1,844) as sensitivity analysis. 11,486 patients remained and the relationship between aspirin use and 28-day (OR = 0.60, 95% CI: 0.50–0.72, *P* < 0.001), 90-day (OR = 0.64, 95% CI: 0.55–0.73, *P* < 0.001), and 1-year mortality (OR = 0.64, 95% CI: 0.57–0.72, *P* < 0.001) remained reliable.

**Figure 3:**
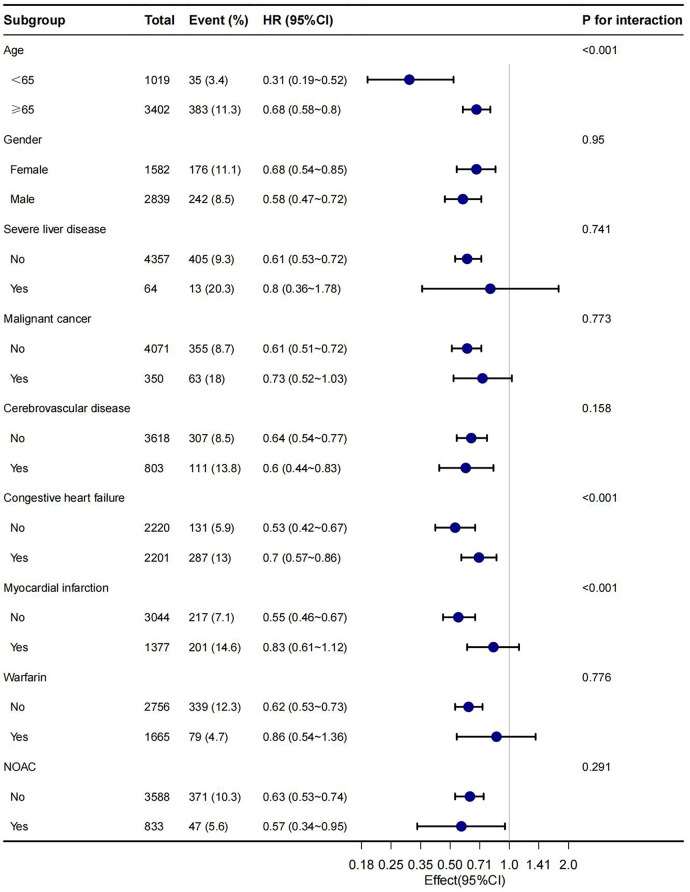
Subgroup analyses of the association between aspirin and 28-day mortality.

**Figure 4:**
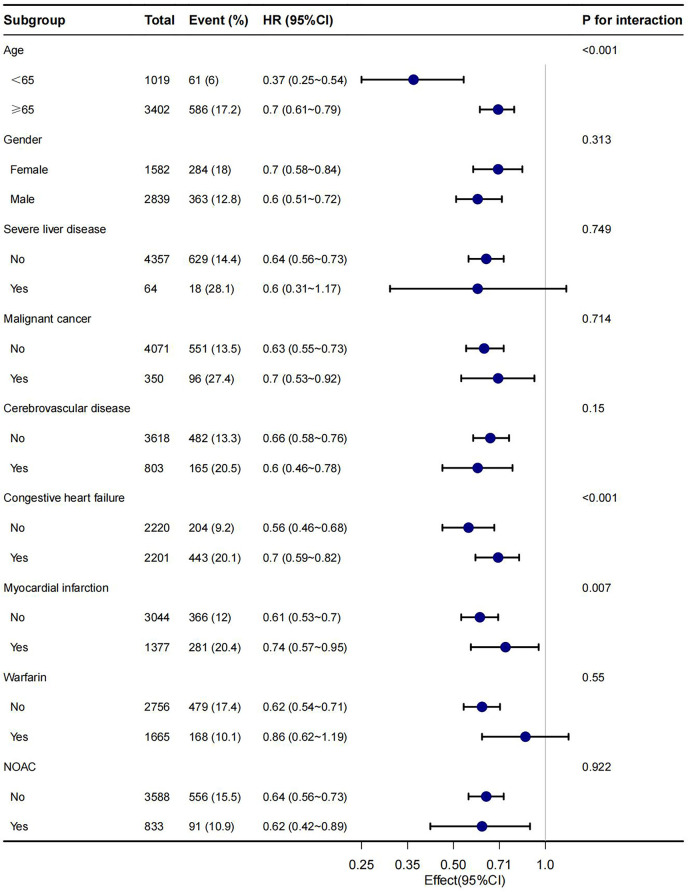
Subgroup analyses of the association between aspirin and 90-day mortality.

**Figure 5:**
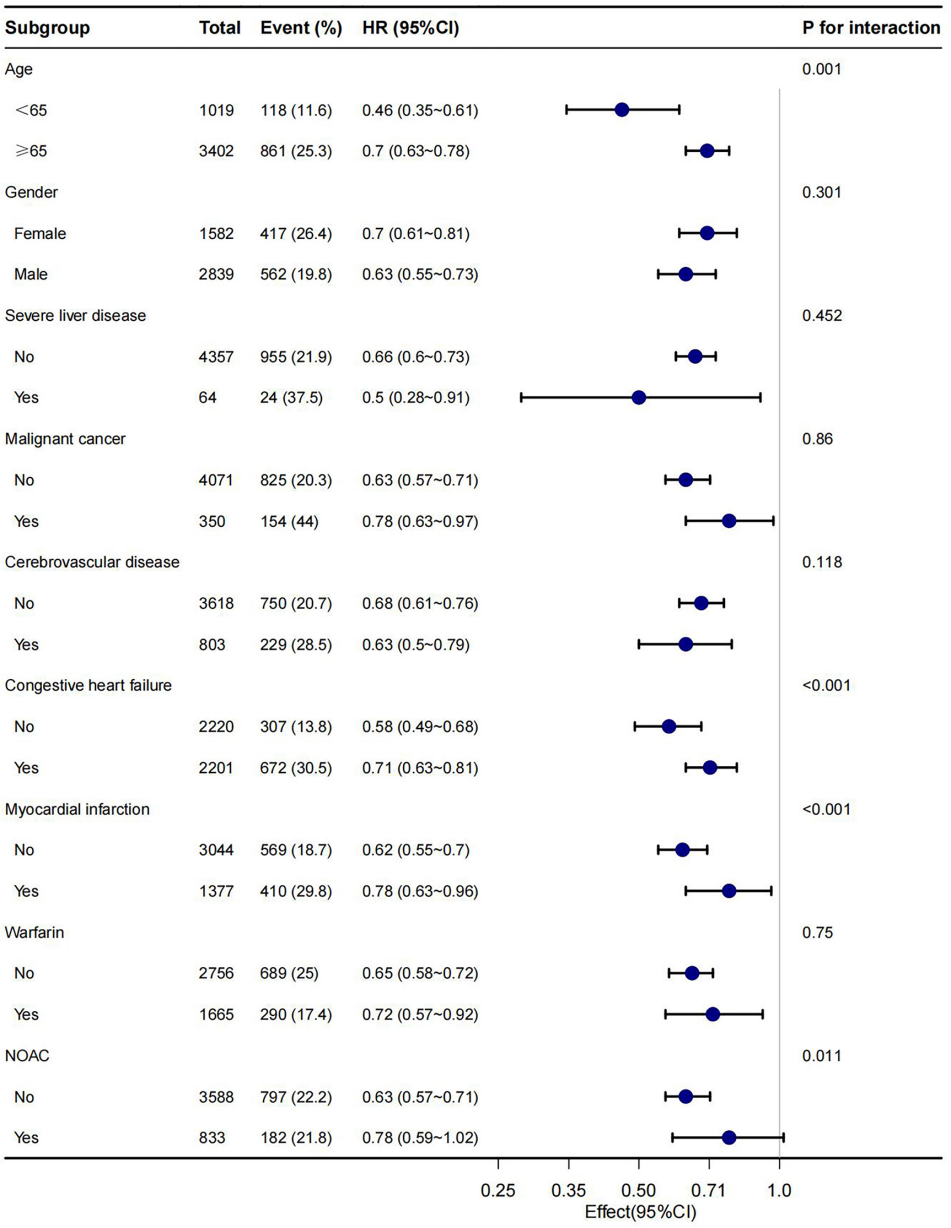
Subgroup analyses of the association between aspirin and 1-year mortality.

In this study, age, gender, malignant cancer, cerebrovascular disease, congestive heart failure, myocardial infarction, warfarin and NOAC were utilized as stratification variables to examine trends in the effects of 28-day, 90-day, and 1-year mortality for these variables (Figures 3–5). It was observed that a limited number of interactions were identified, including age, congestive heart failure, and myocardial infarction (all *P* values for interaction <0.05). The results indicate that a stronger association was detected in individuals aged <65 years old and those without heart failure or myocardial infarction. Irrespective of the concurrent use of anticoagulant medications such as warfarin or NOAC, aspirin was linked to decreased mortality rates, particularly in reducing 1-year mortality, demonstrating superior efficacy when used alone compared to in combination with NOAC.

## Discussion

In this retrospective cohort study, our finding indicated aspirin users with AF hand lower risk-adjusted 28-day, 90-day and 1-year mortality, compared with non-aspirin users. This result suggested an independent association between aspirin and mortality, and additional models confirmed this association. The subgroup analysis of the present study revealed that a more robust association was observed in patients under the age of 65 years and those without a history of congestive heart failure or myocardial infarction.

In the present study, a total of 4,421 out of 13,330 patients (33%) were identified as aspirin users, which represents a higher proportion compared to previous studies. Specifically, Gordon et al. reported that approximately 28% of patients received anti-platelet drugs (15), while another study found that approximately 18.8% of patients received aspirin (16). In contrast, Boyle et al. reported that 28% of individuals received aspirin either prior to hospitalization, during their stay in the intensive care unit, or both (17). The potential source of inconsistency may stem from the definition of aspirin exposure. Specifically, in the MIMIC-IV database, aspirin exposure was operationalized as the documentation of aspirin prescription, whereas Wan-Ting and Lorenzo's cohorts excluded patients who utilized other antiplatelet agents concurrently or transitioned between aspirin and alternative antiplatelet therapies (18).

Critically ill patients with atrial fibrillation exhibit an elevated risk of bleeding as assessed by the HAS-BLED score (19). Consistent with prior study, our findings indicate a comparatively low utilization of anticoagulant therapy, with warfarin prescribed to 16.5% of patients and NOACs to 10.5%. Our results showed that the use of aspirin alone or in combination with anticoagulants was associated with a decrease in all-cause mortality.

According to a meta-analysis conducted by Fangbing Du et al. with a sample size of 22 studies participants, it was suggested that antiplatelet therapy could be beneficial for critically ill patients, particularly those with sepsis (20). The network meta-analysis indicated that aspirin was the most effective antiplatelet therapy. Real-world data obtained from non-valvular atrial fibrillation patients in the United Kingdom demonstrated that the group administered with aspirin exhibited reduced mortality rates compared to those treated with other antiplatelet agents or individuals who did not receive a prescription for atrial fibrillation (10). The administration of aspirin at a dosage of 100 mg per day resulted in a significant decrease in mortality rates among patients in the intensive care unit (15). Similar results were reported by Eisen et al (21). The administration of aspirin prior to surgery has been shown to have a significant impact on decreasing the occurrence of atrial fibrillation in individuals undergoing coronary artery bypass grafting, as well as reducing the length of stay in the intensive care unit (22). Prior studies also demonstrated the efficacy of aspirin in decreasing overall mortality rates among individuals with atrial fibrillation, particularly those with concomitant heart failure, akin to the impact observed with vitamin K antagonists (23).

Nonetheless, there exist certain studies that exhibit incongruity with our results. For instance, Rohan et al. reported that the use of aspirin at baseline was linked to a heightened risk of bleeding and all-cause mortality in individuals with AF (24). Furthermore, a cohort study based on the population demonstrated that the use of aspirin was not correlated with in-hospital mortality during hospitalization for sepsis (25). In a retrospective cohort study conducted across multiple centers, it was observed that critically ill patients with COVID-19 who received aspirin treatment prior to admission and continued with the therapy during their stay in the ICU may have experienced a reduction in mortality rates. However, the initiation of aspirin therapy during hospitalization did not yield similar benefits (16). Additionally, Boyle et al. reported that aspirin therapy administered to ICU patients with acute respiratory distress syndrome (ARDS) either before or during hospitalization was associated with a lower mortality rate (17).

A prior study demonstrated the efficacy of aspirin in reducing mortality rates among patients with sepsis, regardless of the timing of drug administration in relation to the onset of sepsis (11). In our study, we conducted a sensitivity analysis by excluding patients who had been prescribed aspirin prior to admission to the intensive care unit. Despite this exclusion, our findings indicate a consistent relationship between aspirin use and mortality rates.

The present study scrutinized the aforementioned studies that exhibit incongruity with our findings, and posits that the observed disparities may be attributed to several factors. Firstly, the dissimilarities in the research cohorts, comprising critically ill or non-critically ill patients, with varying comorbidities. Secondly, the utilization patterns and timing of aspirin administration. Lastly, the definition of aspirin exposure may have contributed to the observed inconsistency.

The precise mechanism by which the utilization of aspirin is linked to a reduced risk of mortality in individuals with atrial fibrillation remains uncertain. Aspirin acts as an AMPK activation-passing agent to prevent pathological atrial remodeling, reduce fibrosis in AF mice, and prevented the development of atrial fibrillation (9). In cardiac microvascular endothelial cells, aspirin enhances the protective effects of Hsp90 against heat-stressed injury through PI3K-Akt and PKM2 signaling pathways (26). Aspirin mitigates cardiac interstitial fibrosis by impeding the Erk1/2-Serpine2 and P-Akt signaling pathways (27). Procalcitonin level was significantly lower in patients with low-dose acetyl salicylic acid (ASA) indicated that ASA seems to increase the resolution of inflammation (15) which may benefit patients with AF.

The study's subgroup analysis revealed that patients aged 65 years and above, as well as those with congestive heart failure or myocardial infarction, exhibited a weaker association. A recent study demonstrated that administering aspirin treatment to patients with undiagnosed cardiovascular disease prior to hospitalization may result in reduced mortality rates, specifically a significant decrease in 90-day mortality (28). Gordon et al. conducted a retrospective study and described that diagnosis of atherosclerotic vascular diseases did not influence ICU mortality (15).

As an antiplatelet agent, aspirin may lead to increased risk of bleeding. Aspirin administration in critically ill COVID-19 patients may be associated with an increased risk of significant bleeding (16). There was a statistically significant increase in the occurrence of major bleeding and bleeding-related hospitalizations among individuals receiving both oral anticoagulant therapy and aspirin compared to those receiving only oral anticoagulant therapy (29). The correlation between age and the prevalence of cardiovascular disease is widely acknowledged. A prior investigation that examined the tolerability of antiplatelet agents in individuals aged 70 years and above revealed that patients who received aspirin treatment exhibited a notable occurrence of bleeding events when compared to those who received clopidogrel and/or related medications (30). In elderly patients, the benefits of aspirin may be partially offset by its side effects of bleeding. The present study has several limitations that must be acknowledged. Firstly, residual confounding may exist in this retrospective analysis, as is common in such studies. We attempted to mitigate this issue by adjusting for as many potential confounders as possible. Secondly, the study population was limited to ICU patients with AF, thus limiting the generalizability of our findings to non-critically ill patients. Lastly, the results of this study cannot be extrapolated to individuals under the age of 18, as they were excluded from the study. Fourth, as a retrospective study, the causal relationship between aspirin and mortality could not be established. Fifth, Previous studies have shown that BMI, smoking history, and family history of cardiovascular disease were associated with an increased risk of death (31–33), and the inability to adjust for the effects of these factors may lead to an overestimation of the association between aspirin and mortality. Nevertheless, we plan to further explore this issue in future research endeavors. Sixth, Aspirin discontinuation may be warranted as a result of potential side effects such as hemorrhage; however, the bleeding complications experienced by patients in the study were not available to us. Our study encompassed all patients who had used aspirin, including single or short-term prescriptions. Nevertheless, it is worth noting that this inclusion criteria may have underestimated the relationship between aspirin usage and mortality.

## Conclusion

The utilization of aspirin may potentially result in reduced immediate and long-term all-cause mortality among critically ill patients with AF. However, additional randomized controlled trials are necessary to confirm the correlation between aspirin treatment and the outcomes of critically ill patients. Furthermore, we have observed more pronounced effects in patients under the age of 65, without congestive heart failure or myocardial infarction. These associations warrant further exploration.

## Data Availability

The raw data supporting the conclusions of this article will be made available by the authors, without undue reservation.
